# Supplementary material for: A streaming, certificate-based reduction for convex hull preservation in planar point sets
Source: PLoS One. 2026 May 19;21(5):e0349577. doi: 10.1371/journal.pone.0349577 (PMC13186340; doi:10.1371/journal.pone.0349577)
Supplement: S1 Table — Values are reported as mean ± standard deviation over 3 repetitions. Timings were obtained from Python reference implementations on an Apple laptop with an M4 processor and are reported for transparency rather than as hardware-optimised benchmarks. (PDF) [file pone.0349577.s001.pdf]

Supporting Information

Table S1. Absolute runtime (s) on the NYC Taxi dataset under random arrival order

Values are reported as mean ± standard deviation over 3 repetitions. Timings were obtained from Python reference implementations on an Apple laptop with an M4 processor and are reported for transparency rather than as hardware-optimised benchmarks.

n	Full hull (s)	Relaxed filter + hull (s)	Akl–Toussaint + hull (s)
50,000	0.0475 ± 0.0005	0.0327 ± 0.0008	0.0475 ± 0.0006
100,000	0.0974 ± 0.0010	0.0669 ± 0.0022	0.0887 ± 0.0103
250,000	0.2619 ± 0.0015	0.1643 ± 0.0019	0.2072 ± 0.0291
500,000	0.5415 ± 0.0041	0.3320 ± 0.0033	0.4187 ± 0.0554
